# Magnetic-anisotropy modulation in multiferroic heterostructures by ferroelectric domains from first principles

**DOI:** 10.1080/14686996.2024.2391268

**Published:** 2024-08-12

**Authors:** Amran Mahfudh Yatmeidhy, Yoshihiro Gohda

**Affiliations:** Department of Materials Science and Engineering, Tokyo Institute of Technology, Yokohama, Japan

**Keywords:** First-principles calculations, magnetic anisotropy, multiferroic heterostructure, ferroelectric domains, spin-orbit coupling

## Abstract

First-principles calculations incorporating spin-orbit coupling are presented for a multiferroic material as a ferromagnetic/ferroelectric junction. We simulate the interface effect that cannot be described by the single-phase bulk. The in-plane uniaxial magnetic-anisotropy of Co_2_FeSi is observed when the ferroelectric domain is polarized parallel to the interface, whereas the magnetic anisotropy is significantly different in the plane for the electrical polarization perpendicular to the interface. While the single-phase effect dominates the main part of the modulation of the magnetic anisotropy, symmetry breaking due to the interfacial effect is observed in the ferromagnetic ultrathin films. The origin of the modulated magnetic-anisotropy can be attributed to the shifting of specific energy bands in Co_2_FeSi when the ferroelectric domain is modified.

## IMPACT STATEMENT

The origin of strain-induced magnetocrystalline anisotropy in multiferroic Co_2_FeSi/BaTiO_3_(001) heterostructures is clarified by first-principles electron theory. The magnetic anisotropy is modified by interface effects for ultrathin Co_2_FeSi films.

## GRAPHICAL ABSTRACT

**Figure:**
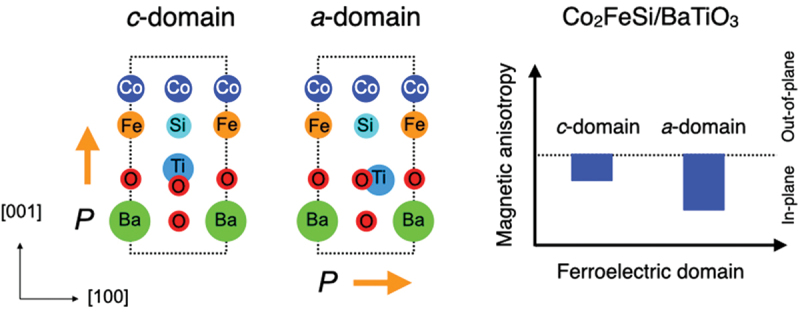

## 1. Introduction

Controlling magnetism via an electric field is an essential and challenging task for realizing fast switching and low power consumption of spintronic devices [1]. For this purpose, multiferroic heterostructures consisting of ferromagnetic (FM) and ferroelectric (FE) materials with the interface magneto-electric (ME) effect have been massively studied due to their capability to easily tune the magnetism by an electric field [2–7]. Various FM candidates have been proposed to achieve high ME coupling coefficients [8–20]. Recently, giant ME coefficients of 10${\;}^{-6}$ to 10${\;}^{-5}$ s/m were observed in multiferroic composites of Co${\;}_{2}$FeSi films with Pb(Mg${\;}_{1/3}$Nb${\;}_{2/3}$)O${\;}_{3}$-PbTiO${\;}_{3}$ (PMN-PT) substrates [21,22]. These are among the highest recorded so far [23–25]. Furthermore, a large ME coefficient in the order of 10${\;}^{-6}$ s/m was also reported in an epitaxial Co${\;}_{2}$FeSi/BaTiO${\;}_{3}$ heterostructure where the FE BaTiO${\;}_{3}$ domain patterns were perfectly transferred to the FM Co${\;}_{2}$FeSi domain structures allowing the strain-mediated magnetic-anisotropy modulation in the Co${\;}_{2}$FeSi films by applying an electric field [26].

Co${\;}_{2}$FeSi is a FM Heusler compound with half-metallic properties where the majority spin band exhibits standard metallicity, while the minority spin band has typical semiconductor behavior [27]. Co${\;}_{2}$FeSi is also known for its high Curie temperature of 1100 K which is suitable for the spintronic technology application [27,28]. The applicability of Co${\;}_{2}$FeSi as the FM component in multiferroic heterostructures was also demonstrated in a recent report where the electric-field control of anisotropic magnetoresistance (AMR) ratio via the so-called FE domain-wall motion was carried out in Co${\;}_{2}$FeSi films grown on top of BaTiO${\;}_{3}$ substrates [29]. Recent first-principles calculations suggested the interface bonding effect plays a significant role in the AMR ratio modulation in Co${\;}_{2}$FeSi/BaTiO${\;}_{3}$ heterostructures [30]. However, the contribution of the strain-transfer effect to the magnetic properties of FM materials in the form of FE-domain modifications has not been discussed in past theoretical studies [16,31–38], even though the experimental works suggested that such effect is the main contributing factor to the ME coupling mechanism in multiferroic heterostructures [39–43].

In this study, using first-principles calculations including spin-orbit coupling (SOC), we investigate the magnetoelastic anisotropy in Co${\;}_{2}$FeSi/BaTiO${\;}_{3}$(001) heterostructures dependent on FE domains to analyze the origin of the ME-coupling mechanism. We calculate the contribution of the magnetic anisotropy from each atomic layer of Co${\;}_{2}$FeSi films. Finally, we clarify the microscopic origin of the anisotropy modulation by using the second-order perturbation method and electronic band-structure analyses. The results reveal that the modulation in the magnetic anisotropy energy in Co${\;}_{2}$FeSi/BaTiO${\;}_{3}$(001) is associated with the shifting of minority-spin bands of the Co atoms around the Γ-point near the Fermi level.

## 2. Computational details

First-principles calculations were performed on the basis of density functional theory (DFT) within the projector augmented wave (PAW) method [44,45], as implemented in the VASP code [46]. Instead of using the PBE functional [47], we use PBEsol, an enhanced functional for solids for describing the exchange-correlation functional with the generalized gradient approximation [48]. The PBEsol can tackle the overestimation of the electric polarization and volume of BaTiO${\;}_{3}$ produced by the PBE functional [38,49]. The calculated lattice parameters (a = 3.97 Å and c = 4.07 Å) and the electric polarization (P = 0.35 C/m${\;}^{2}$) using the PBEsol are close to the experimental findings [50,51]. To describe the structural and electronic properties of Co${\;}_{2}$FeSi, we employ the DFT+U method with $U_{\mathrm{eff}}$ = U – J as 2.5 and 2.6 eV for the Fe and Co 3d states, respectively [28,30,35].

The heterostructure supercells with p(1×1) interface periodicity are constructed by stacking the $1/\sqrt{2}\times 1/\sqrt{2}\times 1$ tetragonal unit cell of Co${\;}_{2}$FeSi(001) layers on the BaTiO${\;}_{3}$(001) as (Co${\;}_{2}$FeSi)${\;}_{8}$–FeSi/TiO${\;}_{2}$–(BaTiO${\;}_{3}$)${\;}_{6}$ and (Co${\;}_{2}$FeSi)${\;}_{9}$–CoCo/TiO${\;}_{2}$–(BaTiO${\;}_{3}$)${\;}_{6}$ for the FeSi/TiO${\;}_{2}$- and CoCo/TiO${\;}_{2}$-terminated interfaces, respectively. We exclude the BaO-terminated interface of BaTiO${\;}_{3}$, as the ME effect only appears in the TiO${\;}_{2}$-terminated interface, which can be partly attributed to its slightly negative charge state [52].

**Figure 1.**
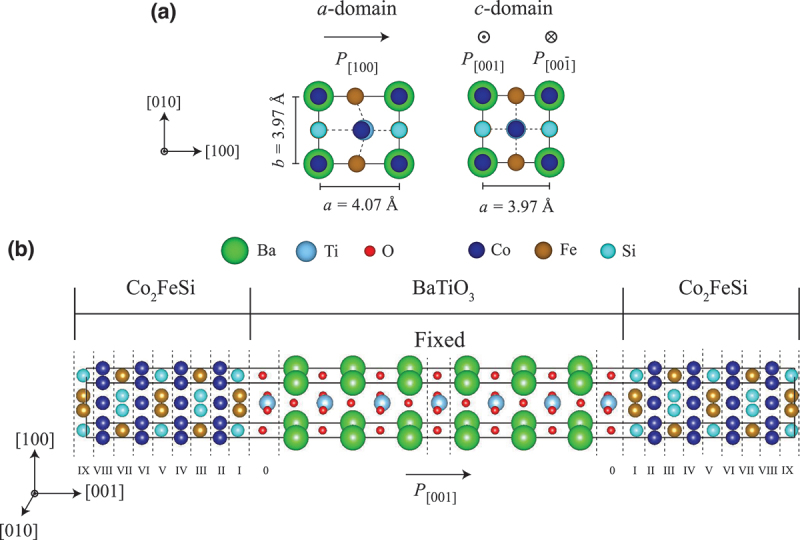
(a) The ferroelectric domain shape for the a and c domains. (b) Schematic Co${\;}_{2}$FeSi/BaTiO${\;}_{3}$(001) heterostructure with the c domain.

We introduce two types of FE domain structures in BaTiO${\;}_{3}$, the a- and c-domains as depicted by Figure 1a which have been experimentally observed [15,26,29,42]. The a-domain has an electric polarization pointing to the in-plane direction ($P_{\left[100\right]}$). As a result, the rectangular-shaped lattice domain with a = 4.07 Å and b = 3.97 Å is formed. On the other hand, due to the out-of-plane electrical polarization ($P_{\left[001\right]}$ and $P_{\left[00\overset{ˉ}{1}\right]}$), the c-domain has square-shaped structure with a = 3.97 Å. It should be noted that the electric polarization of BaTiO${\;}_{3}$ within the heterostructures is difficult to evaluate, because the electric polarization is calculated for the whole unit cell using the Berry-phase method [53–55].

The supercells were relaxed using a Γ-centered k-point grid of 8×8×1 with the cutoff energy of 500 eV, by optimizing the out-of-plane lattice while the in-plane lattice are fixed to those of the calculated bulk BaTiO${\;}_{3}$. The optimized geometries were evaluated using the conjugate-gradient algorithm until the atomic forces on the relaxed atoms were smaller than 0.01 eV/Å, with a total-energy convergence criterion of 10${\;}^{-6}$ eV. During the relaxation, atomic positions of Ti and O atoms at the middle layer are fixed in order to maintain the bulk FE displacements of BaTiO${\;}_{3}$. The relaxed Co${\;}_{2}$FeSi/BaTiO${\;}_{3}$(001) supercell with FeSi/TiO${\;}_{2}$-terminated interface having the c-domain is shown by Figure 1b.

The calculation of magnetocrystalline anisotropy (MCA) energy was carried out by using the OpenMX code with the PBE functional that has higher precision for total energies than PBEsol [56]. Here, the relaxed structures from the VASP calculation were used. The MCA energy ($E_{\mathrm{MCA}}$) per atom was calculated as $E_{\mathrm{MCA}}$ = $E_{\left[100\right]}-E_{\left[001\right]}$ by utilizing the output from collinear calculations employing second-order perturbation theory from first principles, with the 21×21×15 k-point grid [57,58].

## 3. Results and discussions

### Stability of Co${\;}_{2}$FeSi/BaTiO${\;}_{3}$(001) interface termination

3.1.

**Figure 2.**
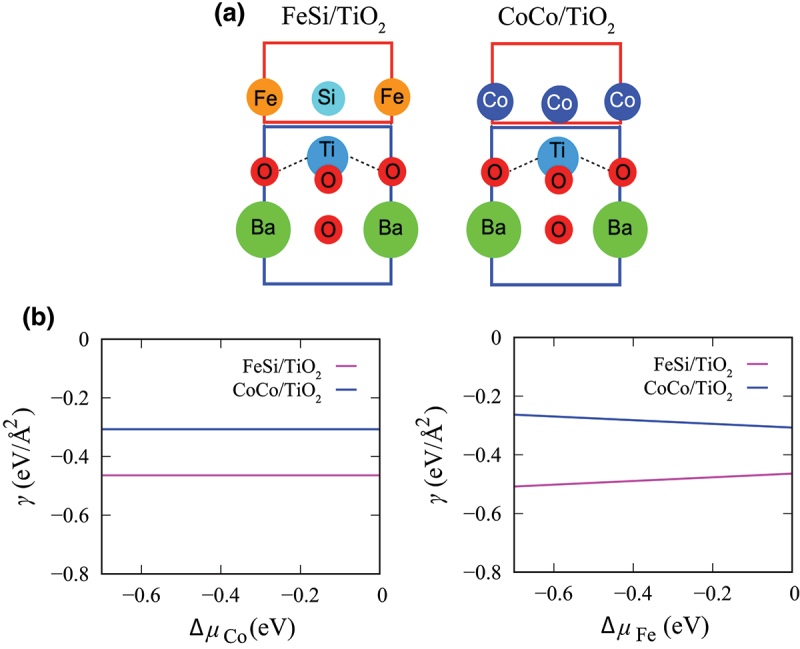
(a) The stoichiometric atomic configurations of $Co_{2}\mathrm{FeSi}/\mathrm{BaTi}O_{3}$ (001) having the $\mathrm{Ti}O_{2}$-termination of $\mathrm{BaTi}O_{3}$: FeSi/TiO${\;}_{2}$ (left side) and CoCo/TiO${\;}_{2}$ (right side). (b) The calculated γ for the FeSi/TiO${\;}_{2}$- and CoCo/TiO${\;}_{2}$-terminated configurations as a function of $\Delta \mu_{\mathrm{Co}}$ and $\Delta \mu_{\mathrm{Fe}}$, respectively.

We first evaluate the interfacial termination of the Co${\;}_{2}$FeSi/BaTiO${\;}_{3}$(001) heterostructure. Here, we examine two possible stoichiometric configurations of the heterostructures to obtain the most stable interface structure (See Figure 2a). For simplicity, the cubic structure of BaTiO${\;}_{3}$ are used. The number of atoms n in material and their respective chemical potentials μ play a significant role in the evaluation of the stability of the interfacial structures. The stability of the interface is evaluated by calculating the formation energy density (γ) of the interface area A using the following formula

(1) $$\begin{array}{rl} & \gamma =\frac{1}{2A}[E_{Co_{2}\mathrm{FeSi}/\mathrm{BaTi}O_{3}}-E_{\mathrm{BaTi}O_{3}}\\ & -(n_{\mathrm{Co}}\mu_{\mathrm{Co}}+n_{\mathrm{Fe}}\mu_{\mathrm{Fe}}+n_{\mathrm{Si}}\mu_{\mathrm{Si}})], \end{array}$$

with the constraint

(2) $$E_{Co_{2}\mathrm{FeSi}}=2\mu_{\mathrm{Co}}+\mu_{\mathrm{Fe}}+\mu_{\mathrm{Si}},$$

where $E_{Co_{2}\mathrm{FeSi}/\mathrm{BaTi}O_{3}}$, $E_{\mathrm{BaTi}O_{3}}$, and $E_{Co_{2}\mathrm{FeSi}}$ are the calculated total energy from the Co${\;}_{2}$FeSi/BaTiO${\;}_{3}$(001) interfaces, clean BaTiO${\;}_{3}$ terminated surface, and bulk Co${\;}_{2}$FeSi, respectively. In addition, $n_{i}$ and $\mu_{i}$ are the number of atoms in the supercell and the chemical potential of an element i. Moreover, the chemical potential of each element must be lower than the energy of an atom in the stable phases of the considered element;

(3) $$\Delta \mu_{\mathrm{Co}}=\mu_{\mathrm{Co}}-E_{\mathrm{hcpCo}},$$

(4) $$\Delta \mu_{\mathrm{Fe}}=\mu_{\mathrm{Fe}}-E_{\mathrm{bccFe}}.$$

Here, $E_{\mathrm{hcpCo}}$ and $E_{\mathrm{bccFe}}$ are the calculated total energy per atom for hcp Co and bcc Fe, respectively. By considering Eqs. (1)∼(4), we can eliminate $\mu_{\mathrm{Si}}$ in Eq. (1) and give the following

(5) $$\gamma =\frac{1}{2A}[\eta -\Delta \mu_{\mathrm{Fe}}(n_{\mathrm{Fe}}-n_{\mathrm{Si}})-\Delta \mu_{\mathrm{Co}}(n_{\mathrm{Co}}-2n_{\mathrm{Si}})],$$

with η is given by

(6) $$\begin{array}{rl} & \eta =E_{Co_{2}\mathrm{FeSi}/\mathrm{BaTi}O_{3}}-E_{\mathrm{BaTi}O_{3}}-n_{\mathrm{Si}}E_{Co_{2}\mathrm{FeSi}}\\ & -E_{\mathrm{bccFe}}(n_{\mathrm{Fe}}-n_{\mathrm{Si}})-E_{\mathrm{hcpCo}}(n_{\mathrm{Co}}-2n_{\mathrm{Si}}), \end{array}$$

the interface formation energy density, γ now can be evaluated as a function of the excess Co and Fe chemical potentials, $\Delta \mu_{\mathrm{Co}}$ and $\Delta \mu_{\mathrm{Fe}}$, respectively, as shown in Figure 2b,c. Negative values of $\Delta \mu_{\mathrm{Co}}$ and $\Delta \mu_{\mathrm{Fe}}$ correspond to Co-poor and Fe-poor conditions, while $\Delta \mu_{\mathrm{Co}}\approx 0$ and $\Delta \mu_{\mathrm{Fe}}\approx 0$ are associated with Co-rich and Fe-rich conditions, respectively.

Figure 2b shows the calculated results of γ for the FeSi/TiO${\;}_{2}$- and CoCo/TiO${\;}_{2}$-terminated configurations as a function of $\Delta \mu_{\mathrm{Co}}$ and $\Delta \mu_{\mathrm{Fe}}$, respectively. Compared to the CoCo/TiO${\;}_{2}$, the FeSi/TiO${\;}_{2}$-terminated interface is more stable in all possible ranges of Fe and Co chemical potentials. At FeSi/TiO${\;}_{2}$, the Si atom of Co${\;}_{2}$FeSi forms a strong covalent bond with the O atom of BaTiO${\;}_{3}$ which seems to have contributed to stabilizing the FeSi/TiO${\;}_{2}$-terminated interface [36]. This can be noticed from the short length of Si−O bond, $d_{\mathrm{Si}-O}$ = 1.78 Å. In contrast, the Co−O bond of CoCo/TiO${\;}_{2}$ has a longer length, $d_{\mathrm{Co}-O}$ = 1.87 Å. Thus, we use FeSi/TiO${\;}_{2}$-configuration for further analyses of $Co_{2}\mathrm{FeSi}/\mathrm{BaTi}O_{3}$(001) heterostructures.

### 3.2. Layer-resolved magnetocrystalline anisotropy energy

**Figure 3.**
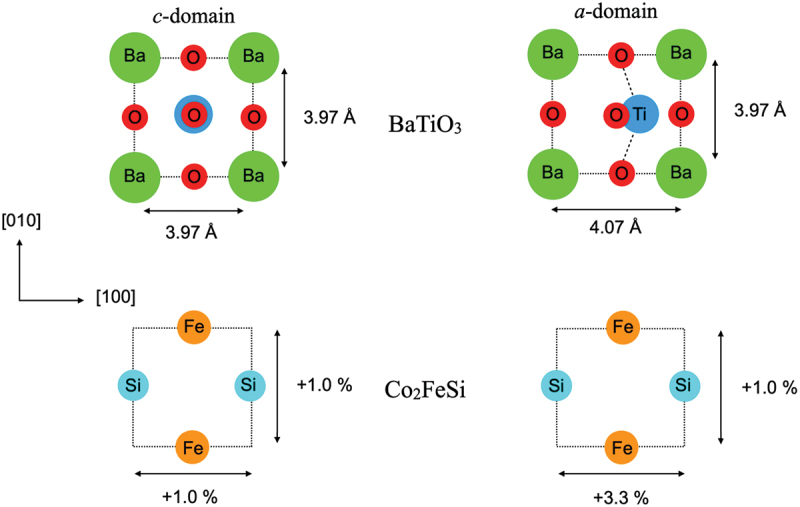
The magnitude of the transferred strain on the Co${\;}_{2}$FeSi film induced by BaTiO${\;}_{3}$ in the a- and c-domains, respectively.

The mismatch between Co${\;}_{2}$FeSi and BaTiO${\;}_{3}$ lattice parameters induces an elongated in-plane strain on the Co${\;}_{2}$FeSi creating tetragonal lattice distortion, which modifies the symmetry and change the magnetic anisotropy of Co${\;}_{2}$FeSi. Figure 3 shows the magnitudes of the in-plane strain on Co${\;}_{2}$FeSi induced by BaTiO${\;}_{3}$, where the strain in the a-domain is larger than that in the c-domain. With this, the magnetic anisotropy modulation in the Co${\;}_{2}$FeSi film of the heterostructure can be induced when BaTiO${\;}_{3}$ lattice domains are modified. In addition, the symmetry breaking at the Co${\;}_{2}$FeSi/BaTiO${\;}_{3}$ interface also induces considerable magnetic anisotropy.

**Figure 4.**
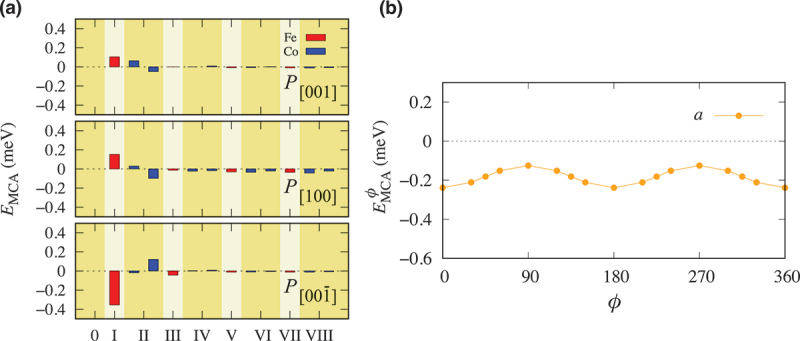
(a) Layer-resolved $E_{\mathrm{MCA}}$ of FeSi/TiO${\;}_{2}$-terminated configuration in the respective a- ($P_{\left[100\right]}$) and c- ($P_{\left[001\right]}$ and $P_{\left[00\overset{ˉ}{1}\right]}$) domains. (b) The polar MCA energy ($E_{MCA}^{\phi }$) as a function of the in-plane angle ϕ from the bulk effect (the atoms occupying thin film layers from the layer III to the layer VIII) in the a- ($P_{\left[100\right]}$) domain.

Here, we first discuss the competition of the magnetic anisotropy among the film layers in Co${\;}_{2}$FeSi of Co${\;}_{2}$FeSi/BaTiO${\;}_{3}$(001) heterostructures. Figure 4a shows the layer-decomposed $E_{\mathrm{MCA}}$ of FeSi/TiO${\;}_{2}$-terminated configuration in the a- ($P_{\left[100\right]}$) and c- ($P_{\left[001\right]}$ and $P_{\left[00\overset{ˉ}{1}\right]}$) domains, respectively. A large $E_{\mathrm{MCA}}$ emerges from the Fe atom at the layer I in both a- ($P_{\left[100\right]}$) and c- ($P_{\left[001\right]}$ and $P_{\left[00\overset{ˉ}{1}\right]}$) domains, respectively, which comes mainly from the magnetic anisotropy due to the inversion symmetry breaking at the interface. Here, we refer to this phenomenon as the *interface effect*. The influence from such effect can also be seen on the layer II.

While $E_{\mathrm{MCA}}$ with $P_{\left[100\right]}$ in the a-domain has perpendicular MCA, $E_{\mathrm{MCA}}$ in the c-domain has a different trend. $P_{\left[001\right]}$ makes MCA perpendicular, in contrast to $P_{\left[00\overset{ˉ}{1}\right]}$ which gives strong in-plane MCA in the c-domain as depicted in Figure 4a. The modified electric polarization, even within the same domain, has strong influence to MCA energy modulation at the interfacial layer as it has previously been demonstrated in ferromagnetic metal films (Fe, Ni, and Co) with BaTiO${\;}_{3}$, Fe with BiFeO${\;}_{3}$, and CoFe${\;}_{4}$N with BaTiO${\;}_{3}$ [18,59–61]. The origin of these phenomena is mainly related to the orbital hybridization between ferromagnetic and ferroelectric atoms at the interface induced by ME effect due to change in electrical polarization. In our study, the interfacial MCA modulation is mainly attributed to the hybridization of d-states between Fe from Co${\;}_{2}$FeSi and Ti from BaTiO${\;}_{3}$.

Due to the range of the interface effect, as short as two monolayers from the interface, we expect this effect is insignificant to the total MCA energy modulation of typical Co${\;}_{2}$FeSi films. However, it should be noticed that further miniaturization of Co${\;}_{2}$FeSi ultrathin films will open up a possibility of the modulation of the MCA energy by the interface effect, as we found the interface effect becomes stronger in the thinner Co${\;}_{2}$FeSi films.

On the other hand, $E_{\mathrm{MCA}}$ from the inner layer atoms (the atoms which occupy the thin flim layers from the layer III to the layer VIII) of Co${\;}_{2}$FeSi film is referred to as the *bulk effect* due to its similarity with the magnetic anisotropy in the bulk counterpart, where the MCA energy in such case is influenced by the induced strain [62]. In our heterostructure, the strain can be induced by the change in the lattice domain such as from the a-domain to the c-domain. To get better perspective, we plot the polar MCA energy $E_{MCA}^{\phi }$ = $E_{\phi }-E_{\left[001\right]}$, where $E_{\phi }$ is the energy with the SOC along the in-plane direction and ϕ = 0 is equivalent to [100] direction. Here, we focus on the contribution of the MCA energy only from the bulk effect. We observed that the calculated $E_{MCA}^{\phi }$ due to the bulk effect of $P_{\left[001\right]}$ is in the same order with that of $P_{\left[00\overset{ˉ}{1}\right]}$, which indicates that the influence of modified electric polarization within the same domain is almost negligible. Therefore, we use $E_{MCA}^{\phi }$ of $P_{\left[001\right]}$ as a representaion for the MCA energy in the c-domain.

Figure 4b shows the contribution of $E_{MCA}^{\phi }$ from the bulk effect for the selected in-plane directions in the a- ($P_{\left[100\right]}$) domain. We can see that $E_{MCA}^{\phi }$ differs for each in-plane direction, indicating that the strain induced MCA is observed in the Co${\;}_{2}$FeSi films. The presence of a uniaxial characteristic of $E_{MCA}^{\phi }$ in the a-domain is related to the larger in-plane strain along the [100] direction (ϕ = 0) as previously shown in Figure 3, which is in agreement with the experimental findings [26,29]. Furthermore, we found the difference of $E_{MCA}^{\phi }$ trends between the a- and c-domains which offers the opportunity of strain-controlled MCA such as by an electric field. Of all the contributed atoms to the bulk effect, the inner layer Co atom plays an important role to the total MCA energy modulation. Thus, further analysis on this particular element will give a complete understanding on the origin of the MCA modulation in Co${\;}_{2}$FeSi/BaTiO${\;}_{3}$(001) heterostructure.

### 3.3. Microscopic origin of magnetocrystalline anisotropy

**Figure 5.**
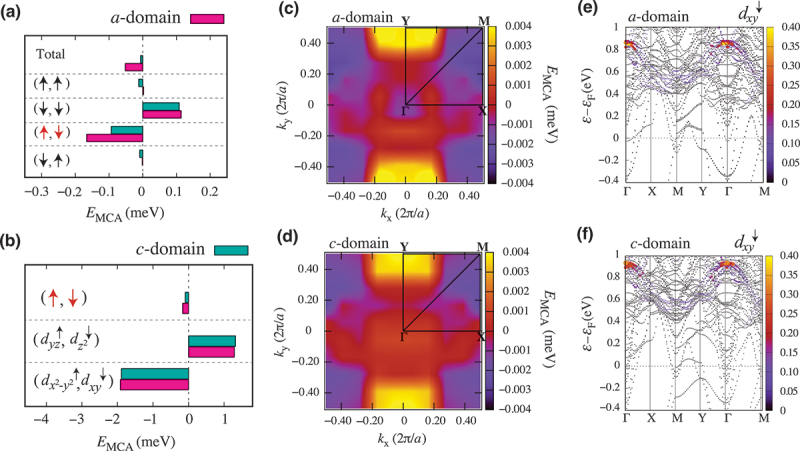
(a) Total MCA energy and four components of the site-decomposed MCA energy (↑,↑), (↓,↓), (↑,↓), and (↓,↓) of the Co atoms (layer VI) in the a- and c-domains, respectively. (b) Selected 3d orbital contributions to the site-projected MCA energy (↑,↓) in the a- and c-domains, respectively. The k-resolved MCA energy distributions of Co atoms (layer VI) in (c) the a- and (d) c-domains, respectively. Orbital-projected band structure of the $d_{xy}^{↓}$ minority spin of Co atoms (layer VI) in (e) the a- and (f) c-domains, respectively.

The microscopic mechanism behind the modulation of magnetic anisotropy in Co${\;}_{2}$FeSi/BaTiO${\;}_{3}$(001) can be understood from the second-order perturbation expression for the MCA energy [57,58,62]. Theoretically, $E_{\mathrm{MCA}}$ can be decomposed into four terms from the viewpoint of spins of unperturbed and virtual states [63,64]. Figure 5a shows total $E_{\mathrm{MCA}}$ and four components of the site-decomposed MCA energy (↑,↑), (↓,↓), (↑,↓), and (↓,↑) of the Co atoms (layer VI) in the a- and c-domains, respectively. We can notice that the modulation of the $E_{\mathrm{MCA}}$ in the Co atoms (layer VI) originates from the spin-flip term (↑,↓) of the MCA energy as shown by the significant increase upon switching of the lattice domain from the a- to c-domains. This finding is similar to the case of the Co atom in the bulk Co${\;}_{2}$FeSi where spin-flip term (↑,↓) in Co is related to the contribution from the quadrupole moment which can be observed via X-ray magnetic circular dichroism (XMCD) and X-ray magnetic linear dichroism (XMLD) measurements [62]. The domain-induced change in the spin-flip term (↑,↓) of the MCA energy of the Co atoms originates primarily from the couplings between the occupied 3$d_{x^{2}-y^{2}}^{↑}$ and unoccupied 3$d_{xy}^{↓}$ states due to the SOC operator $H_{\mathrm{SO}}=\xi L\cdot S$, while the second largest contribution arises from the $\left\langle d_{yz}^{↑}|H_{\mathrm{SO}}|d_{z^{2}}^{↓}\right\rangle$ couplings as shown in Figure 5b. Here, ξ is the SOC constant [65], L and S are the orbital and spin moment operators. Finally, further understanding on the microscopic origin of the MCA energy in the Co atoms (layer VI) can be evaluated by analyzing the in-plane k-dependence of the MCA energy for the a- and c-domains, respectively (see Figure 5c,d). Here, we observe the significant MCA energy modulation around the Γ-point as the lattice domain is modified from the a- to c-domains. The analysis of the electronic band structure of the Co atoms (layer VI) reveals that the changes in the MCA energy around the Γ-point are related to the shifting of the unoccupied minority spin band of the 3$d_{xy}^{↓}$ state (see Figure 5e,f), which is also seen in bulk Co${\;}_{2}$FeSi with lateral strain [62].

## 4. Conclusion

By utilizing first-principles calculations, we evaluated the microscopic origin of the strain-induced MCA energy modulation in the Co_2_FeSi/BaTiO${\;}_{3}$(001) heterostructures with the modification of the ferroelectric domain of BaTiO${\;}_{3}$. We demonstrated that by changing the ferroelectric domain of BaTiO${\;}_{3}$, considerable modulation of the MCA energy in the ferromagnetic Co_2_FeSi was induced. The largest contributions to the MCA energy modulation are related to the bulk effect originating from the inner layer atoms of Co_2_FeSi film where Co is the main contributing factor. Moreover, further investigation using second-order perturbation and electronic band structure analyses revealed that the MCA-energy modulation in Co is associated with the shifting of the unoccupied minority-spin bands near the Fermi level. It should also be noted that the Co_2_FeSi ultrathin film is expected to induce large MCA-energy modulation due to the interface effect. This result is expected to provide the guideline for understanding the fundamental mechanism of the electric-field control of magnetism for future energy-efficient material designs.
